# Supraclinoid direct carotid-cavernous sinus fistula

**DOI:** 10.1259/bjrcr.20170058

**Published:** 2018-03-21

**Authors:** Mohd Firdaus Che Ani, Ramesh Kumar, Mohamad Syafeeq Faeez Md Noh, Ahmad Sobri Muda

**Affiliations:** 1 General Surgery Unit, Faculty of Medicine, Universiti Teknologi MARA, Sungai Buloh, Selangor, Malaysia; 2 Neurosurgery Unit, Universiti Kebangsaan Malaysia (UKM) MedicalCentre, Kuala Lumpur, Malaysia; 3 Department of Imaging, Faculty of Medicine and Health Sciences, Universiti Putra Malaysia, Seri Kembangan, Selangor, Malaysia

## Abstract

Carotid-cavernous fistulas (CCFs) are vascular shunts between the carotid arterial system with direct drainage into the cerebral venous system, mainly to the cavernous sinus. Direct CCF is a well-recognised complication following head trauma. Classically in direct or traumatic CCF, vessel wall tear occurs at the cavernous segment of the internal carotid artery, between the fixed and free segment. Tears at the supraclinoid segment are rare. We report a case of an internal carotid artery supraclinoid segment pseudoaneurysm, with a direct communication with the cavernous sinus, draining into the superior ophthalmic vein.

## INTRODUCTION

Carotid-cavernous fistulas (CCFs) are rare vascular shunts between the carotid arterial system and cerebral venous system, mainly to the cavernous sinus. This permits blood flow from the carotid artery into the cavernous sinus, whether directly or indirectly.^1^ Although rare, it is a well-recognised complication following head trauma.^2^ Traumatic CCF can be classified anatomically as being either direct, with presence of communication between the internal carotid artery (ICA) and the cavernous sinus (Barrow type A), or indirect, where communications exist between dural branches of the internal or external carotid artery and the cavernous sinus (Barrow types B–D).^3^ Typically, in direct or traumatic CCF, the vascular tear occurs at the cavernous segment of the ICA, between the fixed and free segment at the level of cavernous sinus. The usual course of the disease results in an abnormal communication between the ICA and cavernous sinus, presumably due to the shearing effect of the acceleration-deceleration injury, causing carotid segmental tear. Tears at the intracranial segment rarely results in established CCF, as patients will usually suffer traumatic subarachnoid haemorrhage (SAH). Only a few cases have been described in the literature.^4, 5^ We aim to highlight this rarity as well as report such a case encountered in our centre.

##  CASE REPORT

**Figure 1.**
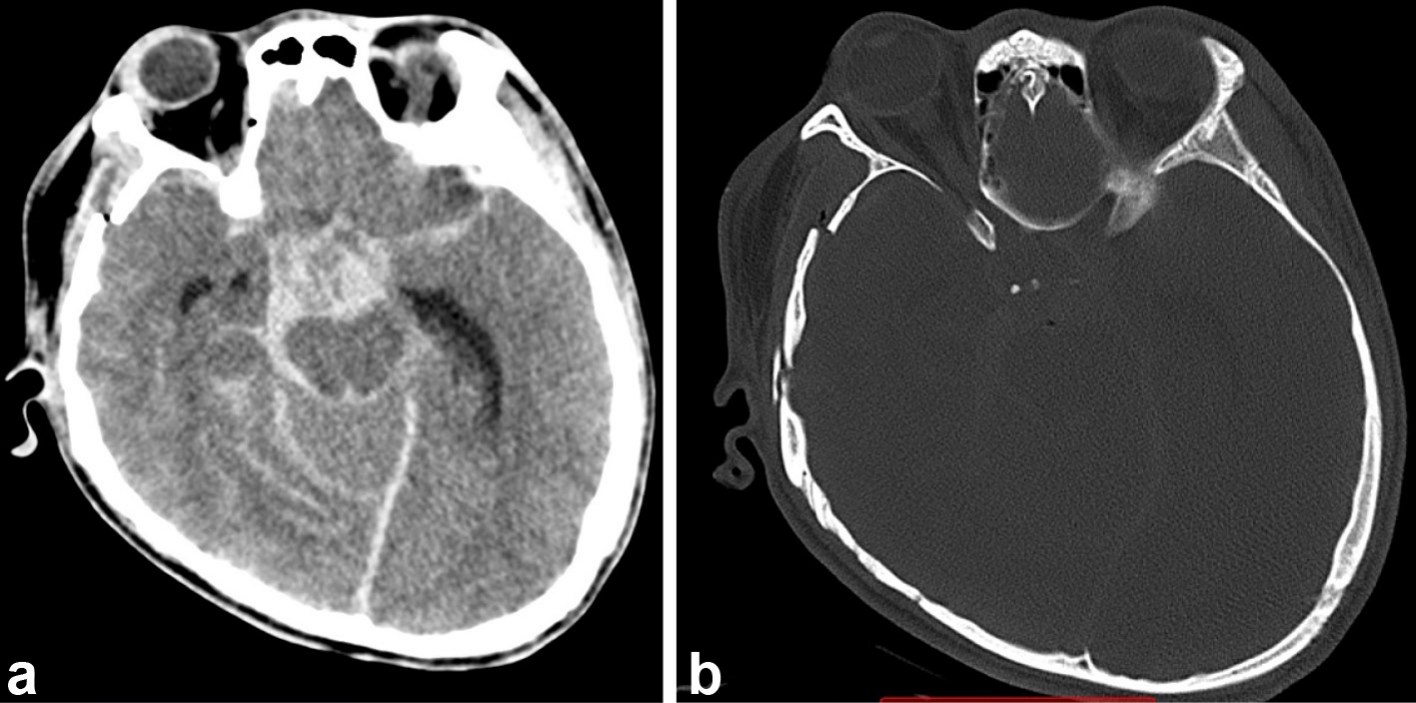
Axial CT images in soft tissue and bone window, at the level of cavernous sinus, showing (a) extensive acute SAH and (b) skull bone fractures that are evident. SAH, subarachnoid haemorrhage.

**Figure 2.**
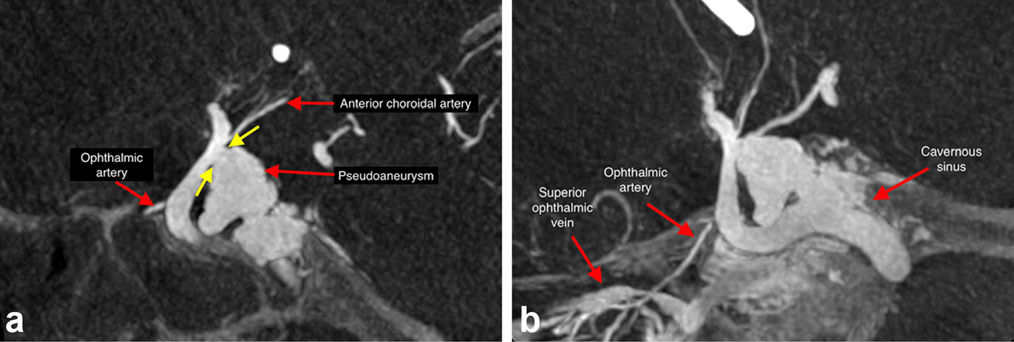
Reconstructed CBCT images, showing (a) reconstructed CBCT image (VasoCT, Philips Medical System) during the diagnostic cerebral angiogram showing the location of right ICA tear with communication between intracranial segment of the ICA and the pseudoaneurysm. Note the neck of the pseudoaneurysm (yellow arrows). (b) Reconstructed CBCT image (VasoCT, Philips Medical System) during the diagnostic cerebral angiogram showing continuation of the pseudoaneurysm into the cavernous sinus and SOV. CBCT, cone beam CT; ICA, internal carotid artery; SOV, superior ophthalmic vein.

**Figure 3.**
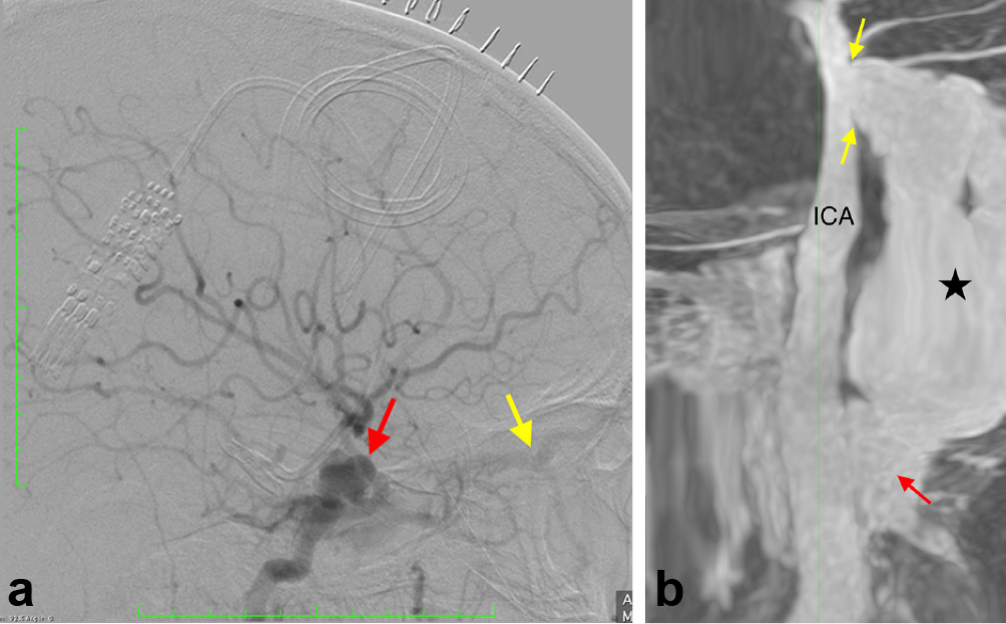
Aangiographic and post processed reconstructed CBCT image, showing (a) right ICA angiographic run (lateral view) showing the aneurysmal vessel (red arrow) and the dilated SOV (yellow arrow). (b) Post processed reconstructed CBCT image showing the neck of the pseudoaneurysm (yellow arrows), in continuation with the pseudoaneurysm (star) and cavernous sinus (red arrow). The ICA is labelled. CBCT, cone beam CT; ICA, internal carotid artery; SOV, superior ophthalmic vein.

A 28-year-old male was brought in by ambulance for an alleged fall from height. He was found to have a Glasgow Coma Scale score of 3/15, following which he was emergently intubated for airway protection and admitted to the intensive care unit for stabilisation. Clinical examination showed traumatic epistaxis, with no suspicion of cerebrospinal fluid leakage. Urgent CT of the brain (trauma series) showed depressed skull fracture with marked SAH (Figure 1a,b), which was evidently disproportionate to the given trauma history. In view of the suspiciously disproportionate SAH, we proceeded with a CT cerebral angiogram. This showed fusiform dilatation of the cavernous, supraclinoid and ophthalmic segment of the right ICA. Medial to the supraclinoid dilatation, a focal saccular outpouching was seen with medial extension into the pituitary fossa. The right cavernous sinus appeared prominent compared to the left. There was additional evidence of a right frontal bone depressed fracture. Collectively, all these findings alerted us to the likely existence of a direct CCF. Confirmatory diagnostic cerebral angiogram was pursued. This revealed a right ICA pseudoaneurysm, suggesting a vascular wall tear, which was seen communicating with the right cavernous sinus and right superior ophthalmic vein (SOV) (Figures 2 and 3). An endovascular intervention was planned, after multidisciplinary team discussion. Unfortunately, the patient’s condition deteriorated further, and passed away after 48 h.

##  DIFFERENTIAL DIAGNOSIS

Dural cavernous fistula

## DISCUSSION

Etiologically, CCFs can be classified as being either traumatic or spontaneous in origin. Of these, traumatic CCFs make up the majority, accounting for up to 75% of all CCFs. The incidence of CCFs in patients with cranio-cerebral trauma has been quoted at 0.2%, while 4% of patients presenting with a basal skull fracture are reported to be afflicted.^1^ Komiyama et al^3^ demonstrated the existence of CCFs as early as a few hours following trauma in their study.

Barrow et al defined four types of CCFs (Types A–D), based on the nomenclature of Peeters and Kroger, with type A being the most common. This connects the ICA directly to the cavernous sinus, often via traumatic rupture of the ICA.^6^ Several pertinent features are suggestive of CCFs; these include SOV dilation, presence of bone fractures, obliteration of the sphenoid sinus, marked enhancement and enlargement of the cavernous sinus and SOV on CT angiography, as well as presence of a pseudoaneurysm.^7, 8^ Many of these imaging features were evident in our case.

Clinical manifestations of CCFs are dependent on a few factors—the size of the fistula, it’s location within the cavernous sinus, flow rate and pattern of drainage. The drainage pattern of CCFs may be either anteriorly or posteriorly.^9^ Of these two patterns, CCFs draining posteriorly into the inferior petrosal sinuses are usually asymptomatic. However, in certain situations when they are clinically symptomatic, they present with facial palsy, trigeminal neuralgia, or ocular motor palsy. In most posteriorly draining cases, clinical signs of orbital congestion are absent unlike those that drain anteriorly. In this clinical entity, symptoms may be as mild as eye redness (unilateral or bilateral), to those suggesting an orbital congestion—proptosis, chemosis and dilated conjunctival vessels.

Our case was both unique and rare in that the tear was shown to occur at the more intracranial segment of the ICA instead of the classically affected cavernous segment. This manifested in the form of a pseudoaneurysm, which communicated likely with the sphenoparietal sinus. We postulated that this eventually lead to retrograde flow in the veins, which drained into the cavernous sinus, especially the superior ophthalmic vein (SOV), due to the high pressure in the carotid system. The resultant pseudoaneurysm possibly traversed the skull base, crossing the dura to flow into the cavernous sinus, hence causing retrograde flow of the affected SOV and cortical veins.

The pathoanatomy of the CCFs influence the choice of endovascular intervention. Commonly, detachable balloons are navigated into the fistula via a trans-arterial route. Trans-venous coiling is also an alternate option. Additionally, other approaches, via direct cannulation of the superior or inferior ophthalmic veins or direct cavernous sinus cannulation via an orbital approach may be used.

In this case, after multidisciplinary meeting with our neurosurgical and neurology colleagues, we decided that the best treatment option was endovascular intervention. Typically, in our centre, treatment of direct CCFs is via detachable balloons; however, noting that the pseudoaneurysm was intracranial in location, support for the detachable balloon might be insufficient. Taking into account the likelihood of difficult navigation from the neck of the pseudoaneurysm into the cavernous sinus, and good visualisation of the inferior petrosal sinus (indicating access), we decided that a combined trans-arterial detachable balloon and trans-venous coiling would be the most effective therapeutic strategy. Unfortunately, patient deteriorated very fast, precluding endovascular intervention.

Open surgery, namely carotid ligation or trapping as well as cavernous sinus exploration, was the routine mode of therapy instituted for CCFs in the old days. Endovascular treatment is the current first line of therapy, with a complete cure rate of more than 80%.^1^ Our patient was planned for endovascular intervention, but unfortunately succumbed due to the severe haemorrhage.

## CONCLUSION

CCFs are uncommon, but a well-known complication following cranio-cerebral trauma. Knowledge of the pathophysiology and relevant imaging features aids in timely recognition, diagnosis and treatment of the disease entity. Although classically affecting the cavernous segment of the ICA, direct CCFs may also arise from the intracranial segment, the incidence of which is not yet known until now–as proven in our experience. Endovascular intervention should be the first line of therapeutic intervention, the potency of which has been clinically proven.

## LEARNING POINTS

1. Carotid-cavernous fistulas, despite uncommon, are known complications following cranio-cerebral trauma. Knowledge of its possible occurrence ensures timely diagnosis and appropriate management, which may prove life-saving.
2. Although classically involving the cavernous part of the ICA, CCF may also rarely involve the supraclinoid segment of the ICA. This was seen in our experience.
3. Endovascular intervention is the first line of therapy, failure of which necessitates open surgery.
